# Rural emergency hospitals: Emerging patterns of adaptation and community perception

**DOI:** 10.1111/jrh.70112

**Published:** 2026-01-16

**Authors:** Anders Van Sandt, Kaitlyn Line, Anja Gruber, Cris Meier, Craig Carpenter, Scott Loveridge

**Affiliations:** ^1^ Department of Agricultural and Resource Economics Colorado State University Fort Collins Colorado USA; ^2^ Department of Social Work Utah State University Logan Utah USA; ^3^ Department of Agricultural and Applied Economics University of Wyoming Laramie Wyoming USA; ^4^ Department of Agricultural, Food, and Resource Economics Michigan State University East Lansing Michigan USA

**Keywords:** health care access, hospital closures, hospital funding, Rural Emergency Hospitals, rural health

## Abstract

**Purpose:**

Rural hospitals face persistent financial challenges that often threaten their survival. To address this, the 2023 “Rural Emergency Hospital” (REH) designation offers Critical Access Hospitals and hospitals with fewer than 50 beds enhanced Medicare reimbursement and annual facility payments if they discontinue inpatient services while maintaining outpatient care and a 24‐hour emergency department. This study evaluates the characteristics of hospitals that choose REH conversion and examines the perceived community impact of the change.

**Methods:**

We analyze Centers for Medicare & Medicaid Services cost report data to compare converting hospitals to eligible nonconverting hospitals. We also conduct a content analysis of 33 news articles and phone interviews with local rural residents to assess how REH conversions are presented in the media and perceived in communities.

**Findings:**

Hospitals that converted to REH status had low inpatient volumes, occupancy rates, and revenues, suggesting they were positioned to benefit financially from eliminating inpatient services. Content analysis revealed that news articles were primarily neutral in tone (54.5%), with most (90%) describing the financial benefits of conversion. Interviews with rural residents highlighted negative perceptions of local health care and revealed that many preferred not to use their local REH even when available.

**Conclusions:**

REH designation may provide financial lifelines to rural hospitals with declining inpatient demand, but community skepticism and limited willingness to use REHs may constrain their role in sustaining health care access. The long‐term effectiveness of this policy may depend on addressing both financial viability and community trust in rural health care delivery.

## INTRODUCTION

The rural health care landscape is rapidly evolving amid persistent financial distress, demographic shifts, and structural pressures. Since 2010, over 140 rural hospitals have closed and nearly 450 more remain at risk,^1^, ^2^ reflecting declining rural inpatient volumes and other systematic challenges.^3^ Although only a small share of the hospitals identified as “at risk” close in any given year, these facilities exhibit similar financial and operational vulnerabilities to those that ultimately close. These closures affect communities well beyond the immediate loss of jobs and revenue, leading to reduced labor force participation and population decline.^4^ More broadly, hospital closures disrupt local health care systems and community vitality, as general practitioners and other skilled professionals are less likely to locate in areas without hospital infrastructure for referrals, coordination, emergency care, and rural livability.

Broader demographic and economic pressures exacerbate these challenges. Many rural counties face ongoing net domestic outmigration,^5^ leaving behind disproportionately older populations—22% of Americans over 65 live in rural counties compared to 15% nationwide.^6^ Aging populations require more specialized care, but hospitals that fail to adapt may face mismatched capacity across departments. Low‐volume rural hospitals are also closing obstetric units faster than urban counterparts,^7^ struggling with insurance, staff recruitment, and reductions in Medicaid payments.^8^ Pandemic relief temporarily alleviated financial distress,^9^ but those funds have expired. Additional pressures include slow adoption of new medical technologies, competition from telehealth, and rising equipment costs.^10^, ^11^ Collectively, these forces contribute to what some observers describe as a rural hospital closure crisis.^2^

In response, policymakers have developed new strategies to support rural health care access. The Rural Emergency Hospital (REH) designation, created under the 2021 Consolidated Appropriations Act, allows small rural hospitals to discontinue inpatient services in exchange for a 5% Medicare reimbursement increase and a $3.2 M annual facility subsidy.* ^12^, ^13^ Eligible hospitals must be located in an Office of Management and Budget‐designated nonmetropolitan county (or county equivalent) and have operated as a Critical Access or acute care hospital with 50 or fewer beds as of December 2020, or be run by a Native American government. Since its implementation, over 40 hospitals have converted to REH status, supported by enabling legislation in several states.† ^14^

The REH model offers a financial lifeline to low‐volume rural hospitals but comes with tradeoffs. REHs must operate a 24/7 emergency department, limit average patient stays to under 24 hours, and meet other participation conditions‡—a tradeoff described by the New York Times as “excruciating” because hospitals may gain financial stability only by giving up inpatient services valued by their community.^13^ REHs forgo eligibility for the Medicare 340B Drug Pricing program (ie, drug discounts) and must establish transfer agreements with Medicare‐certified level I or level II trauma centers, which can be challenging for nonaffiliated hospitals.^13^ REHs cannot provide inpatient services except through distinct skilled nursing facilities, and hospitals that closed before December 27, 2020, cannot reopen under REH status.^15^ While REHs may provide additional outpatient services to meet community needs, these are not eligible for the 5% Medicare reimbursement increase.^15^ While some view these restrictions as burdensome, proponents highlight their potential to preserve emergency access, reduce patient costs, stabilize local economies, and repurpose facilities for other services. Legislative proposals to expand flexibility, including allowances for specialized care and laboratory funding, have yet to advance.^16^

Despite its significance, research on REHs remains limited. The Rural Health Redesign Center identifies potential benefits, including improved access, economic support, preventative care, and cultural awareness.^17^ Early REH counties tend to have higher poverty and uninsurance rates, poorer health, fewer providers, greater emergency department use, and more racial diversity than other rural counties.^1^ Broader rural health research underscores persistent policy misalignments, with federal frameworks often overlooking contextual factors like food insecurity, behavioral health shortages, telehealth access, and long travel distances.^18^ Declining patient volumes have been linked to regional characteristics, Medicare payment type, Medicaid expansion, ownership type, total margin, and population characteristics,^3^ while profitability and competition predict closures.^19^ Conversely, Critical Access status, nonprofit system affiliation, and flexible state regulations provide protection from closure.^19^

Against this backdrop, this study examines the role of REHs in today's rural health landscape. Specifically, we address 3 questions: (1) How are REHs characterized in terms of their financial and operational features? (2) How has the news media portrayed local REH conversions? and (3) How do community members perceive their access to health care and REHs?

## METHODS

This study employs a mixed‐methods, convergent parallel design integrating (1) secondary data on hospital finances, services, and locations; (2) content analysis of newspaper articles; and (3) qualitative interviews of residents local to an REH. § We discuss each of these data collection procedures below.

### Descriptive statistics

We use CMS Hospital Cost Report (HCRIS) data to compare the financial and operational characteristics of REHs and other rural hospital types. We utilize existing code to weight HCRIS cost reports to construct synthetic calendar years for 2000‐2021.^20^ According to the REH program, rural hospitals are those located in areas designated as rural by the Office of Management and Budget's Core Based Statistical Areas. We categorized rural hospitals in the HCRIS data as either (1) eligible but not converted to REH by December 31, 2024; (2) converted to REH in 2023 or 2024; (3) closed during the same period; or (4) all other rural hospitals. Extreme outliers were excluded.|| To supplement these data, we reviewed REH websites to document advertised health services and assessed regional distribution and proximity to neighboring hospitals.

### Content analysis of newspaper articles

We used Nexis Uni, an academic database of a wide range of content, including news, business, and legal materials,^21^ to examine the news media's portrayal and information shared about REHs. Search parameters included: (1) each hospital's name; (2) the term “rural emergency hospital”; and (3) a 2‐year window from January 1, 2022, to December 31, 2024. For hospitals that changed names (n = 2), both versions of the name were searched. The initial search yielded 107 articles. Five articles were dropped along with their duplicates (n = 21) because the content of the articles did not provide specific details about the REH designation specific to a hospital included in the search terms. After removing duplicates of the remaining articles (n = 53), the final sample resulted in 33 relevant and unique articles.

### Interviews of local residents

To examine residents’ perceptions of local health care, and specifically their REH experiences, we conducted semi‐structured interviews during the summer of 2025. Our target population included Extension professionals who worked or lived in counties with operating REHs during the 2‐year period used in the content analysis. Extension professionals operate in a network across a state to deliver the outreach programs of that state's Land Grant university. We utilized Extension professionals because they are deeply engaged in their communities and often have broad perspectives on local needs, services, and institutional trust.

We compiled a list of 145 Extension agents and staff across REH counties using publicly available contact information via Google. From this list, we identified an initial subsample of 29 participants with public contact information representing a range of geographic regions. Recruitment proceeded in 3 waves of emails, totaling 95 outreach emails. The remaining 50 individuals were not contacted because their email addresses were unavailable, or their county or state was already represented in the sample.

Participants completed a brief demographic survey before their interviews, which were conducted with Zoom and recorded with consent. Participants were invited to enter a drawing for a gift card as an incentive. Recruitment continued until no additional responses were received from the initial contact list. While we did not achieve saturation about perceptions of local health care access—reflecting the diversity of perspectives and communities represented—we did achieve saturation for perceptions of local health care quality and perceptions of REHs, with 1 notable outlier. Further sampling might have provided additional insight into our outlier's experience; however, overall, most participants had similar viewpoints. Zoom‐generated transcripts were reviewed by a member of the research team to ensure accuracy.

The semi‐structured interview guide included questions about the participants’ experiences with local health care, perceptions and use of the local REH, and their preferred hospital if faced with a medical emergency. This approach allowed for both comparability across interviews and flexibility to capture unique local insights.

## QUALITATIVE DATA ANALYSIS

This section describes the analytic procedures for both the content analysis of news articles and the qualitative analysis of interviews, complementing the descriptive statistics presented later.

### Coding scheme for content analysis

After finalizing the sample of newspaper articles, 2 team members applied a predetermined coding scheme developed from the Rural Health Information Hub materials and prior literature on REH motivations and challenges.^13^, ^17^, ^22^ The scheme focused on 3 dimensions: (1) overall article tone; (2) reported benefits of REH conversion; and (3) reported drawbacks. Subcodes within each category captured specific details, summarized in Table A1 in the Appendix.

After deriving the coding scheme, 2 research team members independently coded 6 articles to assess interrater reliability. The coding scheme was revised based on this initial coding of articles by having the 2 coders resolve differences in coding. Using the revised coding scheme, the remaining articles were coded. Next, we compiled a coding tracking sheet where each article received either a 1 to indicate presence or 0 to indicate absence for each code. Finally, STATA 19 was used to conduct univariate analysis.

### Interviews

We used a deductive approach to code the interview data.^23^ The interview guide (see Appendix 2 for full interview guide) informed the creation of the codebook, which focused on participants’ experiences with local health care and the REH in their community. Two team members independently coded the same 5 transcripts to identify missing or emergent codes and to establish a threshold of 80% agreement for interrater reliability.^24^, ^25^ Differences were discussed and reconciled, and no new codes emerged, confirming the adequacy of the initial codebook. The remaining transcripts were coded by the 2 research team members who created the codebook.

## RESULTS

The results section is organized into 3 parts: (1) descriptive statistics, (2) content analysis, and (3) interviews.

### Descriptive statistics

We illustrate the long‐standing financial and operational challenges faced by many small rural hospitals using 2000‐2021 HCRIS. Specifically, we observe (1) other non‐REH‐eligible rural hospitals; (2) REH‐eligible hospitals that had not converted as of December 31, 2024; (3) hospitals that obtained REH designation in 2023 or 2024; and (4) hospitals that closed in the same time frame. We compare summary statistics for these 4 groups in Table 1.

**TABLE 1:** Rural hospital summary statistics by type.

Hospital type Definition	Closed Hospital closure in 2023‐2024	REH Conversion to REH in 2023‐2024	REH‐eligible CAH or rural and <50 beds in 2020	Other rural Following OMB definition
Number of hospitals	13	33	1,789	787
Avg. number of beds (2020)	29	25	26	254
CAH designation	47%	39%	75%	0%
*Hospital ownership*				
Government control	15%	48%	38%	16%
Nonprofit control	54%	33%	53%	66%
Private control	31%	18%	8%	18%

*Note*: Table shows 2000‐2021 synthetic calendar year hospital‐level data from CMS Hospital Cost Reports (HCRIS) by rural hospital type.

Compared to other rural hospitals, REH and REH‐eligible hospitals are smaller and more likely to have previously operated as a Critical Access Hospital (CAH). Ownership patterns also differ, with nearly half of REH hospitals operated by local governments, compared to only 16% of other rural hospitals.

**FIGURE 1:**
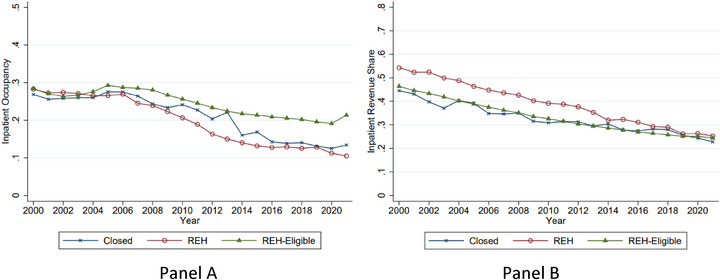
Declining inpatient occupancy rates and inpatient revenue shares. *Note*: Figure shows average inpatient occupancy rates (number of inpatient bed days utilized divided by the total number of available bed days) (panel A) and inpatient share of total patient revenue (inpatient revenue divided by total patient revenue) among hospitals that ultimately closed, converted, or are eligible to convert. Synthetic calendar year hospital‐level data are from the 2000‐2021 CMS Hospital Cost Reports.

The operational pressures facing small rural hospitals are evident in occupancy patterns. As shown in Figure 1, average inpatient occupancy rates (panel A) among hospitals that ultimately converted to REHs declined from about 25% in the early 2000s to about 10% by 2020—a steeper decline than among REH‐eligible hospitals that have not converted. A similar trend appears in the inpatient share of total patient revenue (Figure 1, panel B), which fell more than 50% in 2000 to about 25% by 2020. Together, these measures highlight the erosion of the traditional inpatient hospital model in smaller rural markets.

**FIGURE 2:**
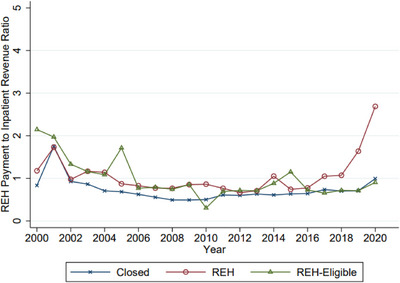
Ratio of fixed REH payments to inpatient revenue. *Note*: Figure shows the average ratio of current fixed annual REH payments ($3,274,392) to the previous years’ inpatient revenue among hospitals that ultimately closed, converted, or are eligible to convert. Synthetic calendar year hospital‐level data are from the 2000‐2021 CMS Hospital Cost Reports.

Consequently, financial viability has shifted with these operational changes. Figure 2 shows that for hospitals converting to REH, the ratio of current fixed REH payments to previous years’ inpatient revenue has risen sharply since 2016, illustrating the financial appeal of the REH model as inpatient volumes declined.

These statistics and trends indicate that REHs represent some of the most resource‐constrained rural hospitals. Their inpatient role has steadily diminished, occupancy rates are relatively low, and financial viability depends increasingly on outpatient and emergency services. The REH designation may, therefore, present an opportunity for resource‐constrained rural hospitals to align operations with a payment model that reflects how they already function.

#### Scope of services and geographic spread

In addition to the HCRIS data, we reviewed the websites of 30 REH facilities to identify available services. Here, we report services mentioned by at least 4 REHs. All REHs advertised emergency room services, and most offered medical imaging (90%, n = 27) and laboratory services (80%, n = 24). Other commonly listed services included rehabilitation/physical therapy (70%, n = 21), primary care clinics (40%, n = 12), surgery (37%, n = 11), and cardiopulmonary care (37%, n = 11). Specialized services such as injection services (27%, n = 8), OB/GYN (23%, n = 7), orthopedic care (23%, n = 7), and respiratory care (17%, n = 5) were less common. Services listed by just 4 (13%) REHs included cancer centers, express care, pain management, sleep services, and pharmacies.¶ Hospital websites are not updated uniformly, and as a result, some services—particularly those offered infrequently—may have been discontinued following REH conversion.

The Sheps Center for Health Services Research at the University of North Carolina maintains a map of REH facilities, showing a concentration in the southern United States, particularly in Arkansas and Mississippi. Fewer REHs are located in the upper Midwest, the Northeast, or the West. Using Google Maps, we recorded driving times between each REH and the nearest hospital (not necessarily a Level II trauma center). The mean driving time was 25 minutes, and the maximum was 82 minutes. Only 2 REHs were located more than an hour from the next closest hospital, while the majority were within 30 minutes of another facility. These patterns suggest that early adopters of REH status tend to be clustered in regions with relatively high rural hospital density and short interfacility travel times.

### Media article content analysis results

Of the 33 coded articles, 15 different REH locations were mentioned, representing 8 states. Among these, 53% were associated with 1‐2 articles, 33% had 3‐5 articles, and only 13% had 6‐8 articles. Articles were first assessed for overall tone—positive, negative, or neutral. Most conveyed a neutral tone (54.5%) describing both positive and negative impacts. Less often, articles had a negative tone (39.4%), with few having a positive tone (6.1%).

Next, we analyzed articles for explanations of why hospitals chose to convert to REH status. Almost all (90.9%) discussed the associated financial benefits, followed by improved chances of survival (63.6%) and solutions to low patient volumes (51.5%). Fewer articles mentioned being able to better support local health outcomes (12.1%) or needed equipment upgrades (9.1%). One hospital further noted that REH designation allowed it to maintain more inpatient capacity than would have been possible under the CAH program, which limits facilities to 25 beds.

Finally, we assessed the concerns hospitals had about becoming an REH. Most articles identified the loss of inpatient services (66.7%) as a primary negative impact of conversion. Other concerns mentioned at similar frequencies included the loss of long‐term care beds (27.3%), reduced inpatient revenue (27.3%), and doubts about whether REH designation would ensure the hospital's long‐term survival (33.3%).

### Interview results

We completed 8 interviews; however, 1 transcript was excluded because the participant worked but did not live in the REH county. The final sample (n = 7) all identified as white, with 1 participant identifying as Hispanic or Latino. A majority of participants identified as women (85.7%, n = 6) and held at least a bachelor's degree (85.7%, n = 6). To ensure anonymity, we do not use participant IDs or report counties, hospitals, or other identifiers, as the limited number of REHs could make participants identifiable. While sufficient for an exploratory analysis of the first REHs, the nonrepresentative and small sample size of the interviewees may limit the generalizability of our findings.

Participants expressed varying levels of confidence in their health care systems, ranging from not at all confident (28.6%, n = 2) to fairly confident (42.8%, n = 3). They were also asked to rate their confidence across 11 service areas (eg, mental health; pediatrics). Confidence was highest in physical therapy/rehabilitation services (fairly confident 57.1% [n = 4] or extremely confident 28.5% [n = 2]), medical imaging (fairly confident 57.1% [n = 4] or extremely confident 14.3% [n = 1]), and general primary care (fairly confident 57.1% [n = 4] or extremely confident 28.5% [n = 2]). Participants had the least confidence in mental health services (not at all confident 14.3% [n = 1] or slightly confident 57.1% [n = 4]), emergency medicine (not at all confident 14.3% [n = 1] or slightly confident 42.9% [n = 3]), orthopedics (not at all confident 71.4% [n = 5]), and neurology (not at all confident 42.8% [n = 3] or slightly confident 28.6% [n = 2]).

#### Local health care access

Participants consistently described health care access as a persistent challenge or barrier, noting that routine and specialized services often required traveling 30‐60 minutes. One participant explained:

[If] you have some kind of ailment or sickness you go to your primary care physician… or if it's something common, a sinus infection, or you know something like that, they'll treat it there [locally]. But anything, you need [like] radiology or X‐rays… Cat scans, you know anything kind of specialized we generally have to go into [Town11], about 30, 45 minutes away.

While the REH was viewed as a partial solution, its limited scope was widely acknowledged, reflecting tension. Some participants expressed gratitude for its convenience and presence in the community, while others remained skeptical about its adequacy and sustainability.

#### REH reputation and use

Participants described the REH's reputation in polarized terms, shaped largely by word‐of‐mouth experiences. Some viewed the facility as an important safety net, particularly for emergencies and people unable to travel. For example, 1 participant said, “Oh, it can be very good, and it can be really bad. It depends on who's on duty at the time that they go in,” while a second participant said, “I think it's very good. It's very highly regarded.” On the other hand, some participants described the REH's reputation as negative, citing poor past experiences and mismanagement that fostered community mistrust. One participant explained:

The hospital has had a really hard time coming back from its past terrible preconceived notions. And every now and then you'll have people that [say] ‘oh, it's not even worth stopping there’, ‘you shouldn't stop there’. But then you have people who having the hospital here has been lifesaving for their family.

Patterns of REH use reflected this reputation divide: residents with fewer resources or mobility constraints were more likely to seek care locally, whereas younger or more mobile populations often bypassed the REH for larger regional hospitals. As 1 participant explained, “In the last 3 years, I know of one person who used it, and it was for an emergency… Otherwise I don't know of anybody who goes there.” Ultimately, the REH's reputation depended less on specific services and more on how experiences circulated within the community.

#### Participant preferences in a health care emergency

Participants were asked which hospital they would choose if they broke a bone or experienced a more serious accident. Most participants distinguished between conditions appropriate for REH care and those requiring transfer. Non‐life‐threatening but urgent problems were seen as suitable for local treatment, while serious trauma or complex needs were expected to be stabilized and referred elsewhere. One participant explained, “I would not choose [REH 1] unless it was like, you know triage. I needed something immediately. But…if I went to [REH 1], I would ask to be transferred.” Participants described this decision as situational: if an injury occurred near the REH, they might use it for triage, but many expressed a preference to transfer as soon as possible to a higher‐level facility. The financial burden of dual billing also influenced decisions to bypass the REH when feasible, as 1 participant explained, “Hospital transfers are very expensive so why would I go somewhere knowing that I'm going to have to get transferred anyway? If I can avoid that bill, I will just drive myself or be transported somehow.”

#### REH designation awareness

Finally, participants were asked if they knew their local hospital had become an REH and what that designation meant. Awareness of the REH designation varied: some participants were familiar with the change, while others were altogether unaware. Among those who knew, most described the shift as largely symbolic, noting that inpatient and specialty services had already been limited for years. As 1 participant said:

I think it has to do with the fact that they don't admit patients, they don't keep anybody overnight, but I don't think they did that before they got this official title, which I could be wrong. But that's really all I know about that. I know that they don't offer any kind of specialty care, but I don't know if it's because of that affiliation, or if it's just because we're extremely rural.

Still, the status carried meaning for how the REH was perceived. Some viewed it as evidence of decline, while others interpreted it as a pragmatic adaptation that focused on what the hospital could reasonably sustain.

I think they've taken the initiative to say this is the best route for us. We may not be able to care long term inpatient for people, you know what I mean, but we are going to I guess put our all into that emergency care to know that hopefully, that's going to be good care that you get there.

For the latter group, the REH was seen as a strategic commitment to provide reliable emergency care, even if broader inpatient services were no longer feasible.

## DISCUSSION

The REH designation represents a policy intervention aimed at stabilizing financially distressed rural hospitals by trading inpatient services for financial survival via enhanced outpatient and emergency care funding. Our analysis demonstrates that hospitals choosing conversion were already operating with steadily declining inpatient volumes and occupancy rates, making the REH model less a strategic pivot than a difficult financial decision that required trading inpatient services for organizational stability. In this sense, REHs appear to be reactive adjustments to long‐standing structural challenges in rural health care markets. Our analysis shows that conversions tend to occur where another hospital is nearby. Given the requirement to transfer patients within 24 hours, future conversions may be limited by the practical difficulty of securing transfer agreements with sometimes remote hospitals. Further, local support for conversion may be constrained if the partner hospital is perceived as too far away. Policy solutions beyond REH‐status may be required to sustain highly isolated rural hospitals.

Our content analysis and interviews also reveal important tensions between institutional survival and community perceptions. While our interview sample was small and not intended to be representative, the insights highlight recurring themes that align with broader patterns across the mixed methods. Although media coverage largely framed the REH program in financial terms—emphasizing its potential to preserve facilities and stabilize budgets—community voices reflected skepticism and persistent dissatisfaction with local health care quality and trust. Many residents indicated they would bypass REHs for nonemergent procedures in favor of larger regional hospitals, underscoring the risk that financial lifelines alone may not restore community confidence or utilization. Even in emergencies, participants often preferred immediately transferring to other hospitals perceived as offering higher‐quality care. To counter these trends, some hospitals may need to pursue a public image and rebranding campaign in tandem with the REH conversion. Public outreach and education could also help mitigate “hospital bypass” behavior,^26^ which contributes to declining patient volumes over time.

Taken together, these findings suggest that while the REH designation may help avert immediate closures, it could also perpetuate deeper challenges related to rural access, quality, and trust unless accompanied by broader reforms. Policymakers and hospital leaders could strengthen the REH model's effectiveness by pairing financial stabilization with sustained community engagement and transparency. The regional clustering of REHs further indicates that the program may be less feasible for highly remote communities, particularly in the Intermountain West, or may function best in states with complementary health care policies. Ultimately, the long‐term viability of the REH program will depend not only on aligning hospital operations with sustainable revenue streams but also on rebuilding trust between rural residents and their local health care institutions.

## CONFLICT OF INTEREST STATEMENT

We, the authors, have nothing to disclose, whether that be personal, commercial, political, governmental, academic, financial, or otherwise. The authors declare no conflicts of interest.

## ENDNOTES

* While REHs must comply with the Medicare Conditions of Participation to receive the fixed monthly payment, the payment is a general subsidy paid out monthly that may be used for fixed costs or services related to Medicare, Medicaid, privately insured, or uninsured patients.

† As of December 19, 2024, the NCSL database included enacted REH legislation from Arkansas, Florida, Illinois, Indiana, Iowa, Kansas, Missouri, Montana, Nebraska, Nevada, New Mexico, New York, Oklahoma, South Dakota, and West Virginia. The NCSL database accessed 2/12/25 at: https://app.powerbi.com/view?r=eyJrIjoiNDNlNTJjNzItNjQyNS00NjVmLTk4MjItZDYzZjBlNTQ0ZDA2IiwidCI6IjM4MmZiOGIwLTRkYzMtNDEwNy04MGJkLTM1OTViMjQzMmZhZSIsImMiOjZ9.

‡ Conditions of participation can be found at: https://www.ecfr.gov/current/title‐42/chapter‐IV/subchapter‐G/part‐485/subpart‐E

§ This study was approved and ruled “exempt” by the Institutional Review Board at Utah State University (IRB: #15301).

|| Extreme outliers include values that were at least 5 times (and up to more than 100 times) larger than the next highest value for a given year and group and hospitals, and also at least 5 times larger than the value for the same hospital in the year prior as we do not believe that these are accurate.

¶ Services mentioned less than 4 times include wound care, pediatrics, neurology, speech therapy, intensive care, mental health/substance abuse (outpatient), hospice, birth center, food & nutrition services, outpatient observation, gastroenterology, podiatry, specialty clinics, ambulance, post‐acute recovery, ophthalmology, care management, infection control, swing bed, home health, transplant, spine, diabetes, endoscopy, inpatient acute care, internal medicine, senior services, urology, ear/nose/throat, outpatient care, and bariatric surgery.

## Appendix

**TABLE A1:** Coding scheme.

Code number	Criteria	Definition
**Attitude of article**		
1	Positive tone about REH (Y/N)	The tone of the article describes the REH designation as positive and/or contributing in a positive way to the health care access of the community.
2	Negative tone about REH (Y/N)	The tone of the article describes the REH designation as negative and/or reducing or harming health care access of the community.
3	Neutral tone about REH (Y/N)	The tone of the article is balanced where the REH designation is presented with both positive and negative attributes becoming an REH.
**Reasons for REH conversion**		
4	Financial benefits (Y/N)	Article describes the financial reasons or benefits that an REH designation has on the hospital.
5	Helps hospital survive (Y/N)	Article describes that the REH designation allowed the hospital to continue its operation opposed to closing.
6	Allows for equipment improvements (Y/N)	Article describes the REH designation allowed the REH to improve or update medical equipment.
7	Hospital has low patient volume	Article describes that prior to the REH designation, there was a low patient volume.
8	Provide support needed for local health outcomes (Y/N)	Article describes because of REH designation, the REH will be able to support certain health outcomes/needs.
9	Hospital was about to keep in‐patient (Y/N)	Article describes that the hospital was able to become an REH and not reduce their inpatient because of CAC or beds under 50.
**Concerns of REH conversion**		
10	Hospital loss of inpatient (Y/N)	Article describes that the designation results in a loss of inpatient services.
11	No longer able to offer long‐term care (Y/N)	Article describes the concern that patients must be transferred after 24 hours. May or may not include discussion of transfers.
12	Loss of revenue because of conversion (Y/N)	Article describes concern of revenue loss from REH designation. Can be any service.
13	REH conversion insufficient for hospital survival (Y/N)	Article describes that the funding offered by an REH might be insufficient for hospital survival.

**TABLE A2:** Interview guide

Main question	Probes
Can you tell me if your friends, family, or even yourself use health care locally?	1. Which type of provider do most people go to locally? 2. For what types of services do the people you know have to leave the community for?
How has the quality of health care in your community changed over the past few years?	1. How about access to health care, how has access to health care in your community changed over the past few years?
When people in your community talk about local health care, what kinds of things do you hear them say?	
How about [name of REH]? Do the people you know use [name of REH]?	1. What types of services do people usually get at [name of REH]?
Thinking more about [name of REH], what is the reputation they have among community members and people you know?	1. Why do you think this is the reputation they have? 2. Can you share examples?
Thinking about yourself and immediate family, if you had an injury, say a broken bone, would you go to [name of REH] or would you go elsewhere?	1. Explain why you would choose [name]? 2. If you had a more serious injury, such as a car accident, and you were able to choose where you went to receive medical care would you go to [name of REH] or would you go [name of the closest hospital]? 3. Explain why you would choose that one?
You might have heard that [name of REH] recently converted to Rural Emergency Hospital status. Did you hear about it?	1. If yes, did you notice any changes to the services at [name of REH]? 2. If no, REH hospitals in most cases give up inpatient beds and focus on emergency room treatment. Patients are expected to be transferred to another hospital after the emergency care. Does this make you more or less likely to go to [name of REH]?
